# Enhanced Recovery Program: Does Preoperative Education Reduces Length of Hospital Stay in Primary Hip and Knee Arthroplasty?

**DOI:** 10.7759/cureus.18639

**Published:** 2021-10-10

**Authors:** Mahmood Ahmad, Hamood Ur Rehman, Aijaz Ali Shaikh

**Affiliations:** 1 Trauma and Orthopedics, University Hospital Waterford, Waterford, IRL; 2 Trauma and Orthopedics, Royal College of Surgeons in Ireland, Dublin, IRL; 3 Trauma and Orthopedics, Our Lady of Lourdes Hospital, Louth, IRL

**Keywords:** educational group (eg), length of hospital stay (los), total hip replacement (thr), total knee replacement (tkr), conventional group (cg), american society of anesthesiologist (asa).

## Abstract

Introduction

Total knee and hip arthroplasty significantly improve the quality of life in people suffering from end-stage hip and knee arthritides. Enhanced recovery programs have shown improved outcomes following elective arthroplasty by decreasing patients’ anxiety, postoperative pain and reducing the length of hospital stay (LOS). The aim of our study is to evaluate the impact of preoperative education programs on LOS in patients undergoing hip and knee arthroplasty.

Methods

Retrospective data was collected from a consecutive series of 520 patients’ charts and the Irish National Orthopaedic Register (INOR) for those who underwent hip or knee arthroplasty from January 1, 2018 to December 31, 2018 in our hospital. The LOS study compared 226 patients in the Educational Group (EG) who attended the Hip & Knee School (Preoperative Education Class) with 294 patients in the Conventional Group (CG) who did not attend the hip and knee educational program.

Results

We identified that LOS decreased to 5.2 days in EG from 5.5 days in CG (p-value equals 0.26, statistically insignificant, t = 1.1093, df = 518), with a mean difference of only 0.3 days (95% CI).

Conclusion

Preoperative education does not reduce the LOS in primary hip and knee arthroplasty.

## Introduction

(*The abstract of this article was presented through poster presentation on 7th March 2020 in Association of Surgeons in Training (ASiT) Annual Meeting in Birmingham, UK. Also, this article's abstract was presented as an oral presentation, to the Surgical Research Society (SRS) Annual Meeting, in the Royal College of Surgeons In Ireland, on 6th Oct 2020*.)

Osteoarthritis (OA) is the most common form of arthritis and the leading cause of chronic disability throughout the world [1]. In western countries, radiographic evidence of this disease is present in 80% of patients by 65 years of age and 60% of these are symptomatic. Approximately 400,000 people in Ireland suffer from OA [2]. The incidence is growing rapidly with the number affected expected to rise further because of the increase in life expectancy and obesity, and the aging population. In Ireland, based on Voluntary Health Insurance, Statistics (VHI), total knee replacements (TKR) have increased in number by 173.4% between 1999 and 2004, whereas total hip arthroplasty (THA) have increased in number by 93% in the same period. [3] TKR and total hip replacements (THR) are some of the most successful medical innovations developed in the last century, which substantially improve patients’ quality of life and have been well validated. 

Hospitals are adapting to the increased demand for joint arthroplasty surgery by developing new and more efficient treatment pathways, the aim of which is to reduce the length of hospital stay (LOS) by early patients discharge in addition to providing continuous care in outside hospital settings like General Physician (GP) follow up [3]. Preoperative education through Hip and Knee Schools is one of the major pathways as part of enhanced recovery programs in patients undergoing THR and TKR, aiming to improve postoperative outcomes (LOS, patients' anxiety, post-op pain, early post-op mobility, etc.) In our regional orthopedic unit, a dedicated Hip and Knee School is providing preoperative education to all patients undergoing Hip & Knee Replacements. Patients booked for hip and knee replacements are advised to attend school before their surgery (optional). A multidisciplinary approach is adopted at the school involving anesthetics, physiotherapists, occupational therapists, orthopedic surgeons as well as arthroplasty nurses (Table 3). The detailed program helps patients to understand the procedure, the in-hospital stay, and the expected outcome to ease their concerns. We request past patients to attend the school to reassure the audience and to provide a more personal experience. An evaluating questionnaire about the participant's experience at hip and knee school is completed to help us improve the program. 

LOS is one of the major contributors to increasing the overall cost as well as waiting list time. Previous studies conducted to assess the effectiveness of preoperative education programs concluded that preoperative education programs are an effective tool for reducing the LOS in addition to alleviating patients’ anxiety, postoperative pain, and early mobility after surgery. The reduction in LOS reduces the overall costs of the surgery and decreases waiting list time for other patients.

Our study helps to identify whether preoperative education programs are effective in reducing LOS or not.

## Materials and methods

**Figure 1:**
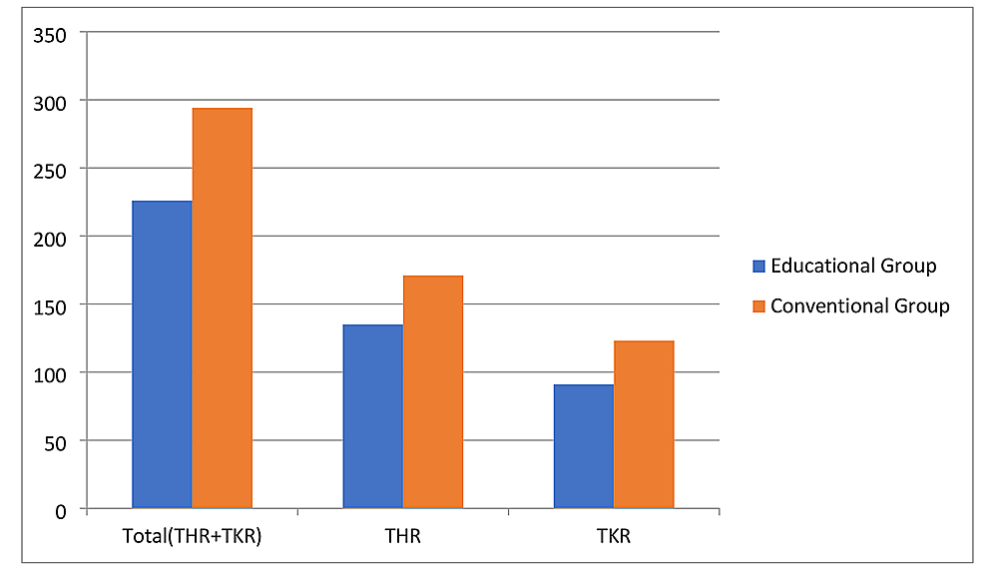
Hip and Knee School attendance 2018 (Y-axis shows the number of patients having TKR or THR in 2018). TKR: Total knee replacement; THR: Total hip replacement.

Retrospective data was collected from a consecutive series of 520 patients’ charts and Irish National Orthopaedic Register (INOR) for those who underwent elective, primary hip and knee arthroplasty from January 1, 2018 to December 31, 2018 in our regional orthopaedic unit (Our Lady's Hospital, County Meath, Ireland). All the data collected, was entered on excel sheet with all the required demographics (patient's attendance of educational classes or not, age range, American Society of Anesthesiologists (ASA) class, TKR vs. THR, complications, cemented vs uncemented THRs, LOS, etc.), collected from patients' charts and INOR. This includes 306 total hips replacements and 214 total knee replacements (Table 1) (Figure 1). 

**Table 1:** Demographics and clinical features of each group educational vs conventional. TKR: Total knee replacement; THR: Total hip replacement.

	Educational Group (Number of Patients)	Conventional Group (Number of Patients)
Age (range)		
<40	3	3
40-65	99	105
66-75	92	118
>75	30	68
Sex		
Male	109	146
Female	117	148
Surgery (number of patients)		
THR and TKR (%)	226 (43%)	294 (57%)
THR	135	171
TKR	91	123
ASA Class (with number of patients in each)		
1.	8	4
2.	157	186
3,	86	47
4.	02	00
Not documented	12	12
General Anesthesia	18	25
Patient shifted to ICU for post-op care (maintaining sats etc not with posts; complications)	18	25
Post-op wound complications	3	3
Post-op Transfusion (In THR vs TKR)		
THR	06	18
TKR	08	11

Patients were broadly classified into two groups for our study purpose:

1) Educational Group (EG) included patients who attended the preoperative education programs (Hip and Knee School), n226 patients (43% of the total cohort). 

2) Conventional Group (CG) included patients who did not attend preoperative education programs (Hip and Knee School), n294 patients (57% of the total cohort). 

All these patients were admitted on the day of surgery (as evident from the date of admission and surgery inpatient notes). Post-operative analgesia was prescribed per ward protocol (paracetamol, OxyContin, and additional oxynorm, as required) and patients were given a ‘day of rest’ after surgery, prior to mobilization. All the patients received thromboprophylaxis (40 mg low molecular weight heparin [LMWH] for three days along with thrombo-embolus deterrent (TED) stocking and foot pumps).

Most patients (477) had a spinal anesthetic (with sedation and regional block) unless it failed or was contraindicated (n43 patients had general anesthesia), per anesthetic notes reviewed for all these patients. Antibiotic prophylaxis was administered preoperatively and for two further intravenous doses of 1.5 g Cefuroxime unless contraindicated (allergy history), as evident from patients' drug kardex.

The patient underwent check X-rays on the 1st postoperative morning, images were reviewed by the Consultant/Registrar, and physiotherapy was started on the first postoperative day for all patients. As per ward protocol, post-op labs/investigations were done on the 2nd postoperative day including C-reactive protein (CRP) (all lab reports were attached in patients' notes).

All THR approaches were either anterolateral or posterior with patients in lateral decubitus position whereas, for TKR, the medial parapatellar approach with patients in a supine position on the table was adapted (mentioned in patients' postoperative notes).

Implants were used according to surgeons' preferences. All the knee replacements were cemented, whereas hip replacements were either cemented or uncemented based on the indication of individual patient bone quality. 

The majority of patients were discharged home after surgery, once fit per criteria (Table 2) with a small number (n37) transferred to convalescence for rehabilitation. This group had an increased hospital stay awaiting a bed in a convalescence home and were excluded from our study (they are not included in our cohort of 520 patients). Six patients had surgical complications post-surgery (wound complications mentioned in patients, progress notes in charts).

**Table 2:** Discharge criteria for the conventional and educational groups.

Transfers from bed to chair and chair to bed independently
• Mobilising safely and independently with appropriate walking aids
• Stair practice completed safely
• Wound is manageable by district nurse and no excessive leakage
• Pain is controlled and manageable
• Straight leg raise with acceptable lag agreed by the consultant

For data analysis and measurement of the required parameters required for our results (average LOS, P-valve, mean difference in two groups, and CI) online statistical analysis app; ''stats kingdom'' was utilized (www.statskingdom.com). we also used SPSS version 24 for the application of the independent T-test and Pearson's chi-square test. The p-value <0.05 was considered significant.

A multidisciplinary approach is adopted at the school involving anesthetics, physiotherapists, occupational therapists, orthopedic surgeons as well as arthroplasty nurses (Table 3). The detailed program helps patients to understand the procedure, the in-hospital stay, and the expected outcome to ease their concerns. We request past patients to attend the school to reassure the audience and to provide a more personal experience. An evaluating questionnaire about the participant's experience at hip and knee school is completed to help us improve the program. 

Ethical approval and consent: not applicable to our study. 

**Table 3:** Hip and Knee School's teaching contents.

Format (Teaching to Patients)
Group education (patients and their family members or friend). Presentation by each member of the multidisciplinary team ( anesthetist, physiotherapist, occupational therapist, arthroplasty nurse, and orthopedic consultant)
Physiotherapist:
In-patient rehabilitation: mobilization within 24 h and increased independence with walking aids. Exercises that can be practiced before and after surgery. Functional aims for discharge: safe and independent mobilization, stairs as required.
Arthroplasty nurse
The patient pathway from admission to discharge. Preparation for surgery: pre-admission assessment, smoking cessation, preparation of the home, and what to bring to the hospital. Methods of anaesthesia, nurse call system, and pain control after surgery. Wound care, optimal hygiene, and protocol for dressing care. Role of the patient in optimizing their recovery; independent mobilization, regular exercise practice, adherence to protocol, and pain management after surgery. Education of patients’ relatives, detailing how they can facilitate recovery. The goal of early discharge after surgery
Orthopedics consultant
Explanation of hip and knee arthroplasty procedures. Risks and benefits of surgery. Response to questions on a one-to-one basis.
Teaching aids

## Results

**Figure 2:**
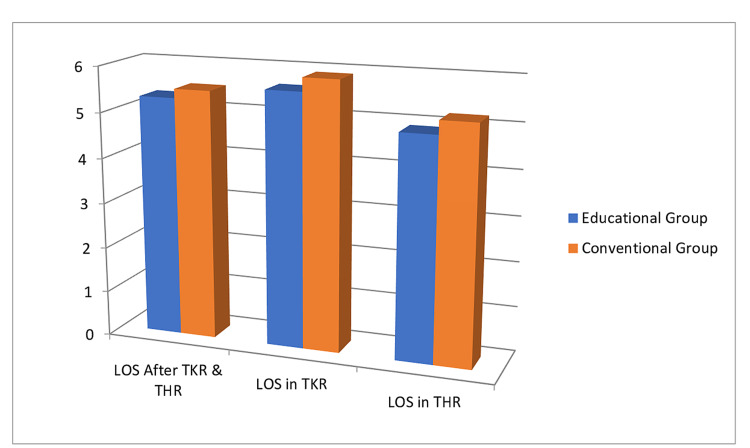
Length of hospital stay (Y-axis shows the number of days stayed in hospital).

From our analysis, the LOS only decreases to 5.2 days in the EG from 5.5 days in CG (p-value of 0.26, which is statistically insignificant), with a mean difference of only 0.3 days (95% CI of this difference: from -0.92 to 0.26) (Figure 2). 

For TKR separately, LOS in EG was 5.6 days compared to 5.9 days in CG (p-value of 0.54 which is insignificant statistically), with a mean difference again 0.3 (95% CI of this difference: from -0.66 to 1.26) (Figure 2). 

Similarly, for THR, LOS in EG was 4.9 days vs. 5.2 days in CG (p-value equals 0.37, statistically insignificant) with a mean difference of 0.3. (95% CI of this difference: from -0.40 to 1.07) (Figure 2). 

**Figure 3:**
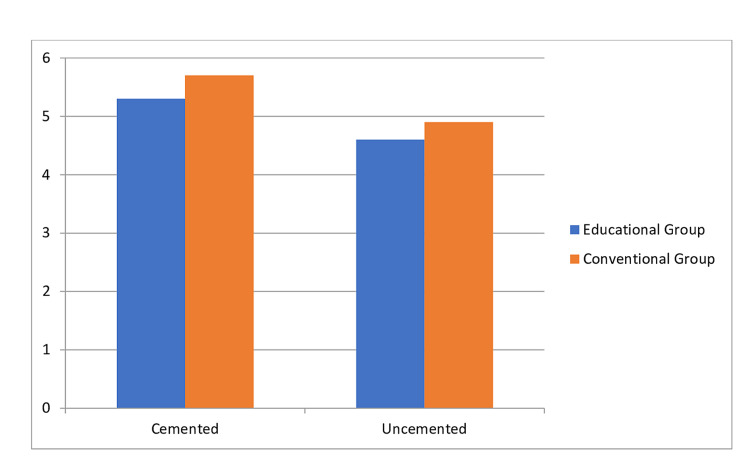
Length of hospital stay in cemented vs uncemented THR (Y-axis shows the length of hospital stay in days). THR: Total hip replacement.

A separate analysis of LOS is performed for cemented vs. uncemented THR. A total of 111 patients underwent cemented THR; LOS was 5.3 days in EG compared to 5.7 days in CG. For uncemented THR, 153 patients were analyzed with LOS of 4.6 days in EG compared to 4.9 days in CG. The mean difference in LOS for both groups was 0.3-0.4 days (Figure 3). 

In our study, a significant increase was seen in LOS with respect to increasing age groups in both CG (p = 0.000) and EG (p = 0.000) as shown in Table 4. Moreover, there was no significant difference in mean LOS in EG and CG (5.22 ± 2.65 vs 5.5 ± 3.8, p = 0.314, respectively). However, the age difference was also insignificant (54.5 ± 19.6 vs 52.9 ± 18.5, p = 0.326, respectively) as shown in Table 5.

**Table 4:** Association between the length of hospital stay and age with respect to interventional groups.

Interventional groups	Age	Length of hospital stay		P-value
		≤7 days	>7 days	
Educational groups	≤40 Years	93 (41.2%)	0 (0%)	0.000
	41-65 Years	60 (26.5%)	1 (0.4%)	
	66-75 years	0 (0%)	34 (15%)	
	>75 years	0 (0%)	38 (16.8%)	
	Total	153 (67.7%)	73 (32.3%)	
Conventional groups	≤40 Years	115 (39.1%)	0 (0%)	0.000
	41-65 Years	66 (22.4%)	0 (0%)	
	66-75 years	0 (0%)	32 (10.9%)	
	>75 years	0 (0%)	81 (27.6%)	
	Total	181 (61.6%)	113 (38.4%)	294 (100%)

**Table 5:** Comparison of length of hospital stay and age in the educational and conventional groups.

Interventional groups	N = 520	Length of hospital stay (days)	P-value
		Mean±SD	
Educational group	226	5.22 ± 2.6	0314
Conventional group	229	5.5 ± 3.8	
Interventional group	N = 520	Age (years)	
Educational group	226	54.5 ± 19.6	0.326
Conventional group	229	52.9 ± 18.5	

## Discussion

Moulton LS et al. study (n882 patients) who underwent primary hip and knee arthroplasty between April 2009 and March 2013, concluded that enhanced recovery programs are effective in reducing the LOS following elective hip and knee arthroplasty. Also, this reduction in LOS was more significant for hip arthroplasty compared to knee replacements [4]. Jones S et al. study collected prospective data on 472 patients, concluded that a pre-operative education program is a safe and effective method of reducing the LOS for knee arthroplasty patients. They found a significant reduction of two days in the LOS in the EG (p-valve: 0.01) [5]. A prospective study by Yoon RS et al. found that the LOS is reduced significantly in patients who underwent hip and knee arthroplasty, after attending preoperative educational programs [6]. O' Reilly M study confirmed previous results that pre-operative educational session remains a very effective way of delivering content to patients regarding their surgery [7-8].

A systemic review by McDonald S et al., to determine whether preoperative education in people undergoing THR or TKR improves postoperative outcomes with respect to pain, function, health-related quality of life, anxiety, the LOS, and the incidence of adverse events (e.g., deep vein thrombosis) concluded that preoperative education does not offer benefits over usual care in terms of reducing anxiety, or in surgical outcomes, such as pain, function and adverse events. Preoperative education may represent a useful adjunct, with low risk of undesirable effects, particularly in certain patients, for example, people with depression, anxiety, or unrealistic expectations, who may respond well to preoperative education that is stratified according to their physical, psychological, and social need [9]. In a systemic review published in Danish journal in 2015 after searching PubMed and Embase (12 randomized studies of preoperative education programs imparted by health professionals to patients were included), found no convincing evidence in favor of preoperative education on outcomes regarding pain, the LOS, patient satisfaction, post-operative complications, mobility, and expectations - except for a significant reduction in preoperative anxiety [10].

Our study does not support the hypothesis that pre-operative education programs (Hip & Knee School classes) help the patients to achieve an earlier discharge with an overall decrease in LOS after the surgery. Patients who attended Hip & Knee School for preoperative education (EG) achieved a decrease in their LOS in the hospital of only 0.3 days (p-value of 0.26) when compared to the CG. This reduction of LOS is clinically insignificant. Our study contradicts the findings of multiple previous studies that proved that hospital stays can be reduced significantly through preoperative educational programs by implementing enhanced recovery protocol. We excluded patients from our study who were awaiting transfer for convalescence post-surgery (n36) as these patients stayed longer in the hospital, although fit to discharge per criteria but staying to get a bed in convalescence/rehabilitation centers. 

In our study, LOS was calculated for TKR and THR individually as well, and the mean difference in LOS in each group (EG and CG) remained the same, 0.3 days, which is statistically insignificant. Similarly, for THR, a comparison is made for cemented vs. uncemented between two groups, which remained the same (0.3-0.4 days difference). All patients were discharged following our discharge criteria.

Our study contradicts most of the previous studies, as discussed in the literature review, that preoperative education can reduce the LOS in addition to patient anxiety and postoperative pain. In most of these studies, the cohort selected was either too small or these were only systemic online reviews, of multiple studies conducted in different parts of the world at a different time, and most of them doesn’t exclude revision arthroplasties from their analyses, which can be a potential reason for making this difference in the hospital stay. Similarly, not even in a single study, after a thorough literature review, a distinction was made for patients who stayed in the hospital for non-medical reasons, which can bring a difference in the final interpretation. 

Our study is supported by Cochrane review, published in 2014, which concluded that pre-operative education offers a minimal benefit over and above standard patient care in reducing LOS after knee or hip joint arthroplasty [9]. Nine studies were included in this Cochrane review. A review in Danish Medical Journal in 2015, reached a similar conclusion that preoperative education is unlikely to reduce the LOS.

## Conclusions

Our study does not support the idea that the implementation of an educational program through the Hip and Knee School is an effective method of reducing the LOS, for patients undergoing elective hip or knee replacement.
